# Where do we stand? The availability and efficacy of diabetes related foot health programs for Aboriginal and Torres Strait Islander Australians: a systematic review

**DOI:** 10.1186/s13047-019-0326-1

**Published:** 2019-03-18

**Authors:** Vivienne Chuter, Matthew West, Fiona Hawke, Angela Searle

**Affiliations:** 1 Faculty of Health and Medicine, School of Health Sciences, University of Newcastle, PO Box 127, Ourimbah, NSW 2258 Australia; 2 Priority Research Centre for Physical Activity and Nutrition, University of Newcastle, PO Box 127, Ourimbah, NSW 2258 Australia

**Keywords:** Aboriginal and Torres Strait islander, Indigenous, Foot, Diabetes, Program, Prevention

## Abstract

**Background:**

Aboriginal and Torres Islander Australians experience considerably higher rates of diabetes and diabetes related foot complications and amputations than non-Indigenous Australians. Therefore there is a need to identify aspects of Aboriginal and Torres Islander focussed foot health programs that have had successful outcomes in reducing diabetes related foot complications. Wider knowledge and implementation of these programs may help reduce the high burden of diabetes related foot disease experienced by Aboriginal and Torres Islander Australians.

**Methods:**

PubMeD, Informit Indigenous collection, CINAHL, SCOPUS, the Cochrane Library and grey literature sources were searched to 28th August 2018. We included any published reports or studies of stand-alone diabetes related foot care interventions, programs, services, educational resources or assessment of these interventions, designed for Aboriginal and Torres Strait Islander Australians.

**Results:**

Thirteen studies detailing interventions in the Northern Territory, New South Wales, Queensland and Western Australia met the inclusion criteria. Five reports described delivery of podiatry services while the other eight investigated educational and training programs. Half of the reports related to aspects of the Indigenous Diabetic Foot program which provides culturally appropriate foot education and training workshops for health care providers. One article reported quantitative data related to clinical patient outcome measures.

**Conclusions:**

No state- or nation-wide foot health programs for prevention of diabetes related foot complications in Aboriginal and Torres Strait Islander Australians were identified. One program achieved high adherence to the national guidelines regarding timing of podiatry review treatments through use of an evidence based foot risk classification tool and provision of services in a culturally appropriate centre.

**Electronic supplementary material:**

The online version of this article (10.1186/s13047-019-0326-1) contains supplementary material, which is available to authorized users.

## Background

Diabetes mellitus is one of the fastest growing chronic diseases in the world [1]. Aboriginal and Torres Islander Australians experience four times the rate of diabetes compared to non-Indigenous Australians, with an overall incidence of 13% [2], and rates as high as 42% reported in some remote communities [3]. As the leading cause of lower limb amputation, and with high rates of associated mortality, diabetes related foot complications are a major but poorly recognised health care burden in Australia, estimated to cost in excess of $1.6 billion annually [4, 5]. Evidence demonstrates Aboriginal and Torres Islander Australians have a three to six fold increased risk of diabetes related foot complications including neuropathy, foot ulcer and lower limb amputation compared to non-Indigenous Australians [6, 7].

Consequently the National Health and Medical Research Council Guidelines for the prevention of foot complications in diabetes state that ‘Until adequately assessed all Aboriginal and Torres Strait Islander people with diabetes are considered to be at high risk of developing foot complications and therefore will require foot checks at every clinical encounter and active follow-up’ [8]. International guidelines suggest that up to 85% of diabetes related amputations could be prevented with early detection of problems and appropriate treatment [9]. Despite the evident need for effective preventative foot care in this population, available data indicate poor engagement with existing preventative care services in contrast to high rates of related hospitalisation and amputation [6, 7, 10].

A number of examples of culturally safe services for Aboriginal and Torres Strait Islander communities in Australia have increased access to combined diabetes care services and improved patient outcomes [11–17]. These share common characteristics, including community consultation in the development, implementation and ongoing management of the service; involvement of Aboriginal Health Workers (AHW); and a focus on self-management and patient participation in health through improved health literacy. There is an obvious and urgent need to identify similarly successful stand-alone foot health programs for the prevention of diabetes related foot complications in Aboriginal and Torres Strait Islander Australians.

## Methods

An electronic database search of PubMeD (using Lit.search https://www.lowitja.org.au/litsearch from the Lowitja Institute which was developed as a search tool for Aboriginal and Torres Strait Islander health articles), Informit Indigenous collection, CINAHL, SCOPUS, and the Cochrane Library was conducted from database inception to 28th August 2018. Additional hand searches of grey literature sources were also conducted including of the Lowitja Institute, Menzies School of Health Research, Australian Indigenous HealthInfoNet (www.healthinfonet.ecu.edu.au), Services for Australian Rural and Remote Allied Health (www.sarrah.org.au), and the Australia Institute of Health and Welfare (http://www.aihw.gov.au/). Reference lists of included studies, clinical guidelines and review articles were also searched. Authors of included studies and reports were contacted where intentions of further evaluation was stated, and, where information was provided, it has been included in this review. The PubMed search strategy as generated from the Lowitja Institute is detailed in Additional file 1. Inclusion criteria were any published reports of stand-alone diabetes related foot care interventions, programs, services, educational resources or assessment of these interventions, designed for Aboriginal and Torres Strait Islander Australians. Interventions were excluded if foot care was embedded within a broader health program due to likelihood of variability in the extent and reporting of the foot care component, and, the confounding effect of the broader health care program on foot specific outcomes. Foot care programs not designed specifically for Aboriginal and Torres Strait Islander Australians were also excluded. One reviewer conducted the electronic searches (AS). Titles and abstracts were independently assessed by two reviewers (AS and VC). Disagreements were resolved by consensus and a third reviewer where necessary (MW).

Extraction of the study data and assessment of the methodological quality of the included studies was conducted by two authors (AS and VC) using the Observational Study and Qualitative Study Appraisal Checklists designed by Health Evidence Bulletins – Wales [18]. These checklists are designed for critical appraisal of observational and qualitative studies and were selected as they include a small number of key domains, are simple checklists rather than scales and were developed using a variety of literature sources [19].

## Results

**Fig. 1:**
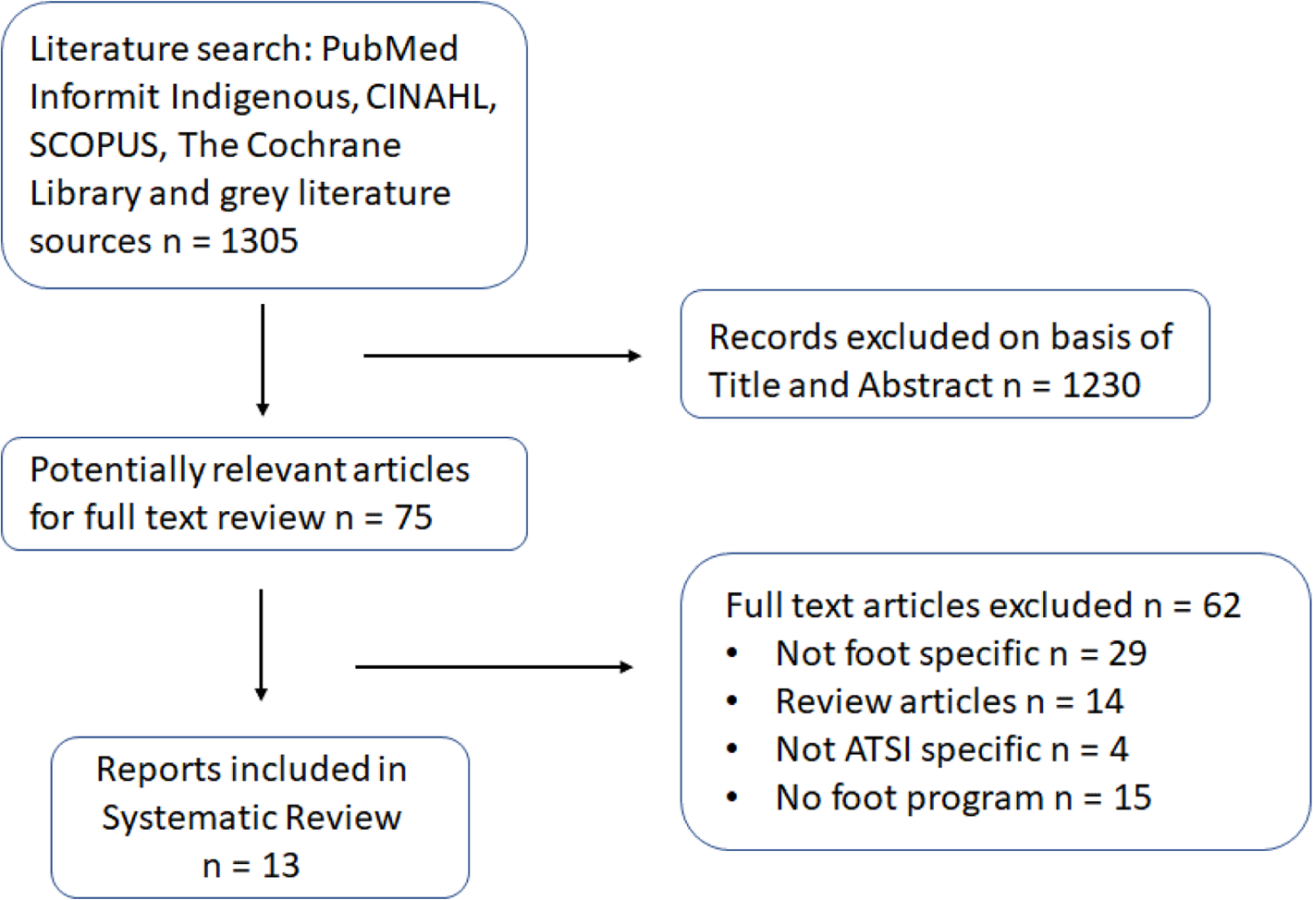
Flow chart diagram of systematic review inclusion and exclusion process. ATSI: Aboriginal and Torres Strait Islander, Combined programs: diabetes or chronic disease programs with a foot health component, review articles: including guideline documents and systematic reviews, no foot program: health interventions without a foot health component

The database and literature search resulted in a total of 1305 citations of which 75 were appropriate for full text review (Fig. 1). After review, 13 articles met the inclusion criteria (Table 1), and 62 studies were excluded (Additional file 2). The methodological quality of the included articles is detailed in Tables 2 and 3. Five articles were reports providing overviews of services provided or materials produced so quality assessment was not considered appropriate [20–24]. Of the remaining articles, two were qualitative studies [25, 26] and six were cohort or cross-sectional studies [27–32]. All of the studies provided detailed information regarding the population studied and aims of the investigations. One of the studies reviewed mainly hospital-based renal dialysis patients, which could make comparison to wider community-based populations difficult [30]. None of the trials reported any cost information related to development or implementation of the interventions.

**Table 1:** Included reports

Author, program & location	Program type	Program description & staffing	Reported outcomes	Culturally safe aspects
Bandaranaike, 2010 [30] Stamping out diabetic foot in the Pilbara, Western Australia Pilbara, WA	Conduct & assess training program	Staff training in IDFP at Port Hedland Hospital Dialysis unit and four Western Desert communities in March–April 2009. Diabetes clients were provided with equipment and taught self-management practices by the program coordinator and staff who attended the workshop. Aims were to: (1) evaluate how the IDFP can be adapted for use in the Pilbara; and (2) evaluate the impact of the program in Aboriginal populations by assessing knowledge and self-management practices pre and post implementation. Program was implemented by a physiotherapist, podiatry student and diabetes educator.	Workshop activities and practical applications rated highly by attendees. Knowledge scores improved post course. Completed DART forms had good inter-rater reliability with podiatrist. Clients reported better access to podiatry services, delivered in a culturally appropriate manner. Clients did not use all of the equipment provided (thongs, soap, mats).	Focus on working within the communities needs and building relationships.
Ballestas 2014 [27] Moorditj Djena (Strong Feet) Perth WA	Service delivery & education	Aboriginal podiatry outreach program. Aim: to identify, manage and prevent foot complications from chronic disease and to improve diabetes self-management. Implemented in 2011 in conjunction with the local Aboriginal community, the Perth Aboriginal Medical Service, and the WA Department of Health. Fixed clinic locations include community centres, an Aboriginal health service, Medicare Locals, a hospital, a health unit office, and a mobile podiatry van. Referrals from GPs, hospitals, community health centres, word of mouth, self and community referrals Staff consists of a coordinator, podiatrists, diabetes educator, AHW.	Attended by 702 clients (by 2013). 14.5% of Aboriginal and Torres Strait Islander adults in region had attended. 3500 occasions of service. High community regard reported. Clinical outcomes evaluation underway with hospital data linkage pre & post enrolment in program.	Community collaboration. Aboriginal staff. Cultural awareness training. Accessible clinics & transport arrangements.
Blatchford 2015 [28] Albury Wodonga Aboriginal Health Service NSW	Service delivery	Podiatry services implemented in an Aboriginal Health service in 2011. Clients had Texas Diabetic Foot Risk evaluation, and incidence of new foot complications recorded. Retrospective audit of clients with Type 2 diabetes for 26 months (*n* = 70). Aim was to identify client’s foot risk status and determine if review appointments met national evidence based timeframe guidelines.	70% attendance at appointments. 94% meet national guidelines for foot assessments.	At ACCHS. Drop in appointments. Transport provided.
Cherbourg Regional Aboriginal and Islander Community Controlled Health Service. Outreach Magazine. [20] QLD	Service delivery & education	Initiation of fly-in podiatry service 20 times/year at ACCHS. Number of Annual Diabetes Neurovascular Foot Assessment. Works with local GP for referrals to vascular, renal or neurological specialists, dietitian, diabetes educator or exercise physiologist. Part of medical students training program.	Reports of lower number of clients requiring treatment of diabetic foot and leg ulcers or amputation dressings. Decrease in number of acute problems. 80 clients consistently attend annual assessment.	At ACCHS. Good community support.
Connors 2008 [29] Who stops the footrot? Interviews with Aboriginal health workers trained in IDFP Regional and remote Qld communities	Assess program	Protocol paper describing two day IDFP workshop followed by telephone questionnaires and evaluation of DART forms. Aim is to determine if the IDFP is an effective method of teaching AHWs how to screen diabetic clients for foot problems and referral onto other health professionals. 15 AHWs participated in the program.	Nil reported to date	IDFP developed for Indigenous clients and staff.
Coombes 2015 [21, 33] A Roving podiatrist North East & West of Alice Springs, NT	Service delivery and education	Development & evaluation of outreach podiatry services for 26 weeks a year, based on the IDFP, in 14 NT communities since 2009. Aim is to maximise availability of podiatry services and upskilling of clinic staff to manage foot problems between podiatrist visits. Services provided by podiatrist and Indigenous podiatry assistant.	Number of clients increased by 167%. Annual foot check percentages increased to 70–90% of community. Increased community engagement. Increased use of protective footwear.	Using IDFP. Male/female team. Indigenous assistant. Emphasis on relationship with communities.
Radowski, 2011 [31] Implementing the Indigenous Diabetic Foot Project in the lower gulf of Australia Rural and remote north-west Qld	Assess training program	A Two-day workshop to train seven AHWs in using the IDFP. Aim is for AHWs to pre-screen diabetic feet, recognise ulcer risk and recognise foot problems that require medical attention or treatment by a podiatrist.	All participants could complete the screening techniques. DART forms now used in assessment of diabetes clients.	Using IDFP.
Schoen 2010 [25] Health promotion resources for Aboriginal people Perth & rural towns, WA	Assess program	A series of six focus group discussions in March–May 2008 with 60 Aboriginal people including Elders, AHWs, community members, and Aboriginal students. Sites included an Elders club in Perth, two rural townships and the Aboriginal Health Training College in Perth. Aim was to determine what materials, medium and foot care messages are preferred by comparison of items produced by IDFP, Healthy Living NT and Derbarl Yerrigan Health Service.	Unanimous support for the IDFP paper-based diabetes foot care education booklet and posters.	Aboriginal reference group provided guidance for the project. Aboriginal and non-Aboriginal interviewer. Reciprocity involving an exchange between the researchers and the participants.
Schoen 2016 [23] Diabetes foot care education movies for Aboriginal people: Bran nue leg & Deadly Kimberley, WA	Develop education program	Documented production of two movies available online or as DVDs. Aim is to improve health literacy and encourage clinic attendance. https://vimeo.com/69131503 https://vimeo.com/85494467 Bran nue leg aimed at people at risk of developing diabetes. Deadly (and not in a good way) is aimed at people living with an amputation. Part of a High Risk foot intervention program.	Initial release so no reported outcomes.	Produced with Goolarri Media Enterprises and local community members.
Townsend, 2012 [32] Evaluation of the NSW Indigenous Diabetic Foot Program for health workers Lower Mid North Coast, NSW	Assess training program	One day IDFP workshop for 11 AHWs with pre, post & 6 month post workshop knowledge questionnaire. Aim was to evaluate the effectiveness of the IDFP by testing AHWs perceptions of the education workshop and integration of the screening tools into clinical practice.	Increase in referrals to podiatrist & knowledge scores post workshop. 55% implemented DART form but no patient workshops run. Occasions of service increased from 7 to 11%.	Using IDFP.
Turner 2006 [22] Podiatry Outreach, Yirrkala Health Centre East Arnhem Land, NT	Service delivery and education	Single day visiting podiatry outreach clinic in April 2005. Aim was to bring a number of health professionals from outside the clinic to address the numerous aspects of diabetes. Presentation on the day provided to clinic staff regarding diabetes foot health, treatment protocols and client education. Staff present included a podiatrist, diabetes educator (for AHWs and clinic staff), nutritionist and nurse. Screening, risk assessment and education provided to Aboriginal community members with diabetes	Collation of diabetes clients onto chronic disease register. Increased awareness of diabetes in the community (not quantifiably measured).	At Aboriginal Health centre. AHWs performing screening Transport to clinic arranged.
Warnock 2004 [24, 35] Indigenous Diabetic Foot Program (IDFP) Palm Island, North Qld	Develop program	Education programs for both AHW and Aboriginal clients with diabetes. Educational card set, videos. Health promotion media and slogans. Components of program include learning: (i) how to care for feet, (ii) how to check feet, (iii) finding pulses on the foot, (iv) using a monofilament (v) understanding the difference between high risk and low risk feet, (vi) how to teach clients the basics of self-care, (vii) completing a DART form, (viii) referral process for a high risk foot. * details combined from a number of sources including presentations and published reports	AHWs more confident in providing education and screening. Increase in AHW knowledge levels up to 6 months post course. Increased referrals from AHWs to podiatrists.	Presented to local focus groups
Watson 2001 [26] Diabetic foot care: developing culturally appropriate educational tools Darwin, East Arnhem, Katherine, NT	Develop education tools	Series of focus groups, telephone calls and mail-outs to help develop a culturally sensitive visual educational tool on foot care for people with diabetes. Attendees included nurses, GPs, AHWs, cross cultural liaison officers and Indigenous Australians with diabetes. Aim is to develop a tool that provides information on control of diabetes, prevention of foot complications and encouragement to seek advice early.	Decision to develop a picture based flip chart. Nil reported to date	In collaboration with Indigenous Australians and health professionals.

*WA* Western Australia, *GP* General Practitioner, *NSW* New South Wales, *ACHHS* Aboriginal Community Controlled Health Services, *%* percentage, *QLD* Queensland, *DVD* digital video disc, *NT* Northern Territory, *AHW* Aboriginal Health Workers, *IDFP* Indigenous Diabetic Foot Program, *DART* Diabetic foot Assessment of Risk Test form

**Table 2:** Quality Assessment of Included Studies - cohort, case-control, cross-sectional studies

Articles	Health Evidence Bulletins - Wales: Questions to assist with the critical appraisal of an observational study eg cohort, case-control, cross-sectional. (Type IV evidence)		Ballestas [27]	Bandaranaike [30]	Blatchford [28]	Connors [29]	Radowski [31]	Townsend [32]
A. What is this paper about?	1. Is the study relevant to the needs of the project?		Y	Y	Y	Y	Y	Y
	2. Does the paper address a clearly focussed issue in terms of:	The population studied?	Y	Y	Y	Y	Y	Y
		(Case-control only) Is the case definition explicit and confirmed?	na	na	na	na	na	na
		The outcomes considered?	Y	Y	Y	Y	Y	Y
		Are the aims of the investigation clearly stated?	Y	Y	Y	Y	Y	Y
B. Do I trust it?	3. Is the choice of study method appropriate?		Y	Y	Y	Y	Y	Y
	4. Is the population studied appropriate?	(Cohort study) Was an appropriate control group used – i.e. were the groups comparable (Case-control study) Were the controls randomly selected from the same population as the cases?	N	N	N	N	N	N
	5. Is confounding and bias considered?	Have all possible explanations of the effects been considered?	N	N	Y	Y	Y	Y
		(Cohort study) Were the assessors blind to the different groups?	na	na	na	na	na	na
		(Cohort study) Could selective drop-out explain the effect?	Y	Y	N	N	N	N
		(Case-control study) How comparable are the cases and controls with respect to confounding factors?	na	na	na	na	na	na
		(Case-control study) Were interventions and other exposures assessed in the same way for cases and controls?	na	na	na	na	na	na
		(Case-control study) Is it possible that overmatching has occurred in that cases and controls were matched on factors related to exposure?	na	na	na	na	na	na
	6. (Cohort study)Was follow up for long enough	Could all likely effects have appeared in the time frame?	Y	N	Y	N	N	Y
		Could the effects be transitory?	N	Y	N	Y	Y	Y
		Was follow up sufficiently complete?	N	N	Y	N	N	N
		Was dose response shown?	na	na	na	na	na	na
C. What did they find?	7. Are tables/graphs labelled and understandable?		na	Y	Y	na	na	Y
	8. Are you confident with the author’s choice and use of statistical methods, if employed?		na	na	Y	na	na	Y
	9. What are the results of this piece of research? Are the author’s conclusions adequately supported by information cited?		Y	N	Y	N	N	Y
D. Are the results relevant locally?	10. Can the results be applied to the local situation? Consider differences between the local and study populations which could affect the relevance of the study		Y	N	Y	N	Y	Y
	11. Were all important outcomes/results considered?		N	N	Y	N	N	Y
	12. Is any cost information provided?		N	N	N	N	N	N
	13. Accept for use as further Type IV evidence?		Y	Y	Y	Y	Y	Y

**Table 3:** Quality Assessment of Included Studies - qualitative studies

Articles	Health Evidence Bulletins - Wales: Additional questions to assist with the critical appraisal of a qualitative study.		Schoen [25]	Watson [26]
A. What is this paper about?	1. Is the study relevant to the needs of the project?		Y	Y
	2. Does the paper address a clearly focussed issue? Are the aims of the investigation clearly stated?		Y	Y
B. Do I trust it?	3. Is the choice of a qualitative method appropriate?	What was this study exploring (eg behaviour/reasoning/beliefs)?	Y	Y
		Do you think a quantitative approach could have equally/better addressed this issue?	N	N
	4. Was the author’s position clearly stated?	Has the researcher described his/her perspective?	Y	N
		Has the researcher examined his/her role, potential bias and influence?	Y	N
	5. Was the sampling strategy clearly described and justified?	Check to see whether: • the method of sampling is stated or described • the investigators sampled the most useful or productive range of individuals and settings relevant to their question • the characteristics of those included in the study are defined (and are comparable to the wider population)	Y	Y
	6. Was there an adequate description of the method of data collection given?	• Is the method of data collection described and justified? • How the data were collected (eg audiotape/videotape/field notes)? • If interviews were used, were the questions pre-tested? • If observation was used, is the context described and were observations made in a variety of circumstances?	Y	N
	7. Were the procedures for data analysis / interpretation described and justified?	Check to see whether: • a description is given of how the themes and concepts were identified in the data • the analysis was performed by more than one researcher • negative/discrepant results were taken into account • the data were fed back to the participants for comment	Y	Y
C. What did they find?	8. What are the primary findings?	Consider whether the results: • address the research question • are likely to be clinically important	Y	Y
	9. Are the results credible?	Were sequences from the original data presented (eg quotations) and were these fairly selected? • Is it possible to determine the source of the data presented (eg numbering of extracts)? • How much of the information collected is available for independent assessment? • Are the explanations for the results plausible and coherent? • Are the results of the study compared with those from other studies?	N	N
D. Are the results relevant locally?	10. Can the results be applied to the local situation?	Consider differences between the local and study populations (eg cultural, geographical, ethical) which could affect the relevance of the study.	Y	Y
	11. Were all important outcomes/results considered?		Y	Y
	12. Accept for further use?		Y	Y

The thirteen articles detailed foot programs which were conducted in New South Wales (NSW), Queensland (QLD), Western Australia (WA) and the Northern Territory (NT) with the majority in rural, regional or remote areas (Table 1). Five of the articles described delivery of podiatry services to Aboriginal communities [20–22, 27, 28, 33], three described the development of specific foot education resources [23, 24, 26], and the remaining five assessed foot educational and training programs [25, 29–32]. Seven of the thirteen reports described aspects of the Indigenous Diabetic Foot Program (IDFP) [21, 24, 25, 29–32].

### Delivery of podiatry services

Moorditj Djena is an Aboriginal podiatry outreach program implemented in 2011 in metropolitan Perth, WA, which was initially funded as part of the Australian Federal Government’s ‘Closing the Gap’ program [27, 34]. The program’s aim is to identify, manage and prevent foot complications and to improve diabetes self-management. Culturally secure treatment is offered in community venues as well as two customised mobile vans. Staff include AHWs, podiatrists and diabetes educators. An initial review of the program describes the number of clients seen, occasions of service, percentage of local Aboriginal and Torres Strait Islander clients with diabetes seen by the clinic (14.5%), and staff perceptions of the program.

One study outlines the findings from a retrospective 26 month (2012 to 2014) clinical audit undertaken at the Albury-Wodonga Aboriginal Health Service (AHS) in NSW. [28] Podiatry services only commenced at the health service in 2011 and the audit’s aim was to determine if evidence based standards for podiatry services based on the patients risk classification were being met. In the sample population (*n* = 729) a high rate (94%) of adherence to the national guidelines regarding podiatric review timeframes was found. The authors suggested that the excellent outcomes may be due to provision of services according to national guidelines, in a culturally safe manner, alongside flexible arrangements such as drop in appointments and access to transport services [28].

The establishment of podiatry outreach services in remote communities is described by three reports [20–22]. The Yirrkala Health Centre in East Arnhem Land NT, organised an inaugural Diabetes Day in 2005 to coincide with a visit from a podiatrist and AHW specialising in diabetes education [22]. Clients received podiatry services on the day and were registered for recall according to their risk status, and both staff and clients received diabetes related foot education. Another two reports detail the establishment of visiting podiatry services (for 26 weeks a year) to fourteen remote communities to the east and west of Alice Springs NT [21, 33]. The program, which started in 2009, is based on the IDFP and is funded by the NT Primary Healthcare Network’s (PHN) Medical Outreach Indigenous Chronic Disease Program. A podiatrist and an Aboriginal podiatry assistant provide general podiatry services, foot health checks, and education in foot first aid, diabetic foot care and footwear. Between 2014 and 2017, activity report data from the NT PHN Outreach Services demonstrated the number of patients receiving podiatric care increased by 167% [33]. The increased attendance rate is anecdotally credited to the close relationships the team built with each community, in conjunction with a personalised and targeted approach to encouraging people to attend clinics and self-manage their foot health. A similar visiting podiatry service was established by the Cherbourg Regional Aboriginal and Islander Community Controlled Health Service in QLD, with funding provided by the Australian Government Rural Health Outreach Fund [20]. The podiatrist visits twenty times a year and also encourages attendance for an annual diabetes neurovascular foot assessment. The program is supported by the medical community with the local general practitioner (GP) providing follow-up care and referrals to specialists as required.

### Development of foot educational resources

The Indigenous Diabetic Foot Program (IDFP) is the most widely used template for delivery of diabetes related podiatry services to Aboriginal and Torres Strait Islander Australians. The IDFP was developed in QLD in 2005 to provide culturally appropriate foot education for Aboriginal and Torres Strait Islander Australians and training workshops for AHWs and podiatrists regarding diabetic foot screening [24, 35]. The education resources developed for clients are mostly visual aids (posters, a CD ROM, videos and an educational card set), which feature Aboriginal feet and stories. Resources for AHWs and podiatrists include a Diabetic Foot Assessment of Risk (DART) form, a self-care education model for use with clients, and advice on referral pathways if required. The DART form requires assessment of the foot (pulses, sensation, foot lesions and deformities, amputations or scars), assessment of client self-care practices (awareness of the need for foot care, wearing footwear, and ability for self-care), assessment of an overall risk classification and a date for future foot review.

The development of culturally sensitive visual educational resources for Aboriginal and Torres Strait Islander Australians are described by two studies [23, 26]. Two diabetes foot care movies (‘Bran nue leg’ and ‘Deadly (and not in a good way)’) were produced as part of a larger High Risk Foot intervention in WA [23]. An Aboriginal media company and local Kimberly community members were involved in production of the movies, ensuring the message was delivered in a culturally sensitive manner. The aim was to improve health literacy related to at-risk feet, and encourage early presentation to health services for foot problems by Aboriginal Australians in the Kimberley. The report described the recent development and release of the movies and we did not identify any follow up report of the evaluation or effectiveness of the movies. Another study details the processes behind the creation of a foot care educational tool for Aboriginal and Torres Strait Islander Australians in the NT [26]. The preferred educational resource, a graphical flip-chart, was determined following a workshop and a series of focus groups with health professionals (including general practitioners, nurses, and AHWs) and Aboriginal and Torres Strait Islander Australians with diabetes. Again, the study was published prior to the release of the materials, and assessment of the effectiveness of the tool was not identified in this review.

### Assessment of foot educational and training programs

Assessment of aspects of the IDFP is described by five reports [25, 29–32]. One study conducted focus groups with 60 Aboriginal Elders, health workers, students and nurses, to determine their preferred messages and media for communication of diabetes foot care information [25]. The paper-based resources produced by the IDFP, with photographs of feet of Aboriginal and Torres Strait Islander Australians, were the participants’ favoured option.

The other four studies describe training and evaluation of AHWs knowledge and competency when using the IDFP [29–32]. One report described a protocol for a two-day IDFP workshop in QLD to teach AHWs how to screen for diabetes related foot problems. Follow up investigations included telephone questionnaires with AHWs, examination of completed DART forms and comparison of completed DART forms versus number of people in the community with diabetes [29]. No published assessment of the workshop was identified during this search. Another report describes the evaluation of the IDFP in a hospital site and four remote communities in the Pilbara WA [30]. Workshop attendees completed a workshop evaluation form and DART forms completed in the hospital were cross-checked by a podiatrist. Community members were assessed regarding their perceptions of the course as well as knowledge and self-care for diabetic feet. Feedback suggested that the program was culturally safe and improvements were seen in community member’s knowledge and self-care pre and post course. An assessment of AHWs perceptions of the IDFP following a one day course and integration of the tools into practice was also conducted on the Lower Mid Coast of NSW [32]. The participants had higher knowledge and confidence levels immediately after and six months after the workshop. Just over half (55%) of participants implemented the DART screening form into clinical practice, however none had implemented a ‘Look after your feet workshop’, which is one of the patient education components of the IDFP. While an increase in the number of Aboriginal and Torres Strait Islander people attending the podiatrist was also reported, this could not be attributed to the effects of the training as the number of referrals from course attendees was not tracked. The final report describes a two-day IDFP workshop run in the Lower Gulf area in QLD to train AHWs in screening diabetic feet [31]. Following the training, the AHWs implemented the DART form as a standard for all clients with diabetes.

## Discussion

The aim of this review was to systematically evaluate the current literature to determine the availability and effectiveness of stand-alone foot health programs for the prevention of diabetes related foot complications in Aboriginal and Torres Strait Islander Australians.

While national and state governments have developed general Aboriginal and Torres Strait Islander health plans and strategies, no state- or nation-wide foot health programs for prevention of diabetes related foot complications were identified in the literature. Benefits of existing foot care programs for Aboriginal and Torres Strait Islander peoples are therefore largely restricted to the area/s in which they are delivered. Of the thirteen reports that met the inclusion criteria (Table 1), one detailed quantitative data related to clinical patient outcome measures following implementation of the program [28]. The most widely used program, the IDFP (Indigenous Diabetic Foot Program), has been assessed with regard to workshop attendee competency and client perceptions of the educational material provided. Evaluation of the IDFP’s impact in terms of patient outcomes and rates of diabetes related foot complications is yet to be undertaken/published [35].

Despite years of research, Aboriginal and Torres Strait Islander Australians still have worse health outcomes than non-Indigenous Australians. A key criticism of the research to date, both in Australia and in Indigenous populations of other countries, is a high concentration of descriptive research regarding populations, risks and measures, rather than a focus on assessment of the efficacy of interventions to close the health outcome gap [36, 37]. To enable an intervention to be successfully and widely implemented by front-line clinicians it must meet rigorously designed methodological standards, it must have been tested for its effectiveness and reproducibility, and it must be easily accessible in peer reviewed literature [36]. While six of thirteen reports (46%) in this review do describe interventions, only one (8%) of the reports [28] describes patient based clinical outcomes following implementation of a podiatry service. Overall, methodological quality of the studies that were eligible to be assessed was mixed. Lack of robust assessment of outcome data, including comparison to control data in relation to patient outcomes, was common to all studies. In addition, reporting of economic analysis related to development or implementation of the interventions was not provided in any study. Lack of documented follow-up evaluation of interventions, similar to that seen in the stand-alone foot programs in this review, has been described previously. A report investigating the implementation of 1082 Australian Indigenous health, cultural and education programs, found only 8% of programs were evaluated [38]. Additionally, in contrast to non-Indigenous health interventions, the majority of the reports included in this review were published in non-peer reviewed grey literature. These factors make it challenging for clinicians and researchers to locate and implement best practice evidence in relation to foot health programs for the prevention of diabetes related foot complications in Aboriginal and Torres Strait Islander Australians.

Although assessment of clinical outcomes following implementation of foot health strategies has not been commonplace in the past that seems to be changing. The current lack of evidence regarding successful components of Aboriginal and Torres Strait Islander focussed foot health programs in reducing diabetes related foot complications has been clearly recognised by those working in clinical areas. Programs and assessments that are currently underway have incorporated clinical and cost outcomes as part of their implementation plans. In WA the Moorditj Djena program [27] has recently conducted an internal review, the results of which will be published upon completion (private correspondence). The review includes a data linkage project to examine participant outcomes pre and post enrolment in the program. In particular, they examined the population reach of the program, the reach in high-risk settings, occasions of service versus comorbidity, and the number and duration of hospitalisations for diabetes-related conditions. NSW Health is currently in the planning stages of a Healthy Deadly Foot initiative (private correspondence). It has the potential to be the largest foot health program for Aboriginal and Torres Strait Islander Australians implemented to date. One metropolitan local health district (Central Coast), three rural and regional local health districts (Hunter New England, Illawarra Shoalhaven, Western NSW), and one speciality network (St. Vincent’s Health Network) have committed to the project. Goals include the development of AHW roles in local health districts and Aboriginal communities, delivery of appropriate cultural and clinical support, encouraging more Aboriginal and Torres Strait Islander people to train as podiatrists and an evaluation strategy to examine the outcomes of the project. In addition, in 2018, the authors of this report embedded an Aboriginal and Torres Strait Islander foot health clinic into the undergraduate podiatry program at the University of Newcastle. The clinic, led by an Aboriginal podiatrist and AHW, provides prevention and management services for diabetic foot complications for Aboriginal and Torres Strait Islander Australians in the local community. It also provides clinical placement to all undergraduate podiatry students as a mechanism to increase cultural awareness in the future podiatry workforce. The service is currently undergoing clinical and educational outcome evaluation for peer-reviewed publication. This includes evaluation of service utilisation with historical control data for the broader clinical service, effectiveness of diabetes education for improving client knowledge of diabetes self-care, and post-placement changes in self-perceived confidence in provision of culturally safe care in undergraduate students.

The results of this review should be viewed in light of several limitations. Although this review was designed to be comprehensive with a robust search on relevant databases, the search strategy may not have located all Australian health initiatives involving diabetes related foot disease specifically delivered for Aboriginal and Torres Strait Islander Australians. Many of these reports are located in grey literature sources and structured search engines are not available. Additionally, this review only describes programs where reports regarding the development, implementation, or effectiveness of initiatives were publically available. It is likely that programs exist that have not been published in any form; this is particularly probable where these involve individual practitioners, or are in small service delivery models.

## Conclusions

No state- or nation-wide foot health programs for prevention of diabetes related foot complications in Aboriginal and Torres Strait Islander Australians were identified by this review. One report, a clinical audit of podiatry services offered at a NSW AHS, provided data supporting the clinical effectiveness of the program. The authors describe a high rate of adherence to the national guidelines regarding timing of podiatry appointments which may be related to classification of patients according to evidence based risk status, and provision of services in a culturally safe manner. More data regarding aspects of successful Aboriginal and Torres Strait Islander foot programs should soon become available with planning and assessment of programs already underway in both WA and NSW.

## Additional files

Additional file 1: PubMed search strategy as generated from the Lowitja Institute. (DOCX 28 kb)

Additional file 2: Excluded studies. (DOCX 13 kb)

## Abbreviations

ACCHS: Aboriginal Community Controlled Health Services
AHS: Aboriginal health service
AHW: Aboriginal health worker
DART: Diabetic Foot Assessment of Risk form
DM: Diabetes mellitus
GP: General Practitioner
IDFP: Indigenous diabetic foot program
NSW: New South Wales
NT: Northern Territory
QLD: Queensland
WA: Western Australia
