# Supplementary material for: Where do we stand? The availability and efficacy of diabetes related foot health programs for Aboriginal and Torres Strait Islander Australians: a systematic review
Source: J Foot Ankle Res. 2019 Mar 18;12:17. doi: 10.1186/s13047-019-0326-1 (PMC6423788; doi:10.1186/s13047-019-0326-1)
Supplement: Supplementary file 1 — PubMed search strategy as generated from the Lowitja Institute. (DOCX 28 kb) [file 13047_2019_326_MOESM1_ESM.docx]

**Additional file 1:** PubMed search strategy as generated from the Lowitja Institute

((Diabetes mellitus[mh] AND (((australia[mh] OR australia*[tiab]) AND (oceanic ancestry group[mh] OR aborigin*[tiab] OR indigenous[tw])) OR (torres strait* islander*[tiab])) AND medline[sb]) OR ((Diabet*[tiab] OR ((type 1[tiab] OR type I[tiab] OR type 2[tiab] OR type II[tiab]) AND diabet*[tiab]) OR gestational diabet*[tiab] OR prediabet*[tiab] OR pre diabet*[tiab]) AND ((((au[ad] OR australia*[ad] OR australia*[tiab] OR northern territory[tiab] OR northern territory[ad] OR tasmania[tiab] OR tasmania[ad] OR new south wales[tiab] OR new south wales[ad] OR victoria[tiab] OR victoria[ad] OR queensland[tiab] OR queensland[ad]) AND (aborigin*[tiab] OR indigenous[tiab])) OR (torres strait* islander*[tiab])) NOT medline[sb]))) AND (program OR strategy OR plan OR promotion OR course OR educat* OR treatment OR intervention OR therapy OR improve* OR clinic OR workshop OR manage* OR project)
