# Supplementary material for: Where do we stand? The availability and efficacy of diabetes related foot health programs for Aboriginal and Torres Strait Islander Australians: a systematic review
Source: J Foot Ankle Res. 2019 Mar 18;12:17. doi: 10.1186/s13047-019-0326-1 (PMC6423788; doi:10.1186/s13047-019-0326-1)
Supplement: Supplementary file 2 — Excluded studies. (DOCX 13 kb) [file 13047_2019_326_MOESM2_ESM.docx]

**Additional file 2: Excluded studies**

| **Author** | **Title** | **Description** | **Type** |
| --- | --- | --- | --- |
| Collaborative for Healthcare  Analysis and Statistical Modelling (CHASM), The University of Western Sydney. First Edition: Fourth Iteration December 2017 | High Risk Foot: Geographical Inequities, Importance of Different Diagnosis Groups, Forecast Hospitalisations, and  Access to Services | Analysis and predictions regarding high risk foot admissions in WA to inform clinical service planning and service evaluation for podiatry services. | Review |
| Bailie, R. S., et al. (2004) | A multifaceted health-service intervention in remote Aboriginal communities: 3-year follow-up of the impact on diabetes care. Med J Aust 181(4): 195-200 | Examine the trends in processes of diabetes care and in participant outcomes after an intervention in two remote regions of Australia. Including foot sensation, reflexes, pressure and infections. | Not foot specific |
| Bailie, R., et al. (2007) | Improving organisational systems for diabetes care in Australian Indigenous communities. BMC Health Serv Res 7: 67. | Two annual cycles of assessment, feedback workshops, action planning, and implementation of system changes in 12 Indigenous community health centres. Including 3 monthly foot checks. | Not foot specific |
| Bergin, S. M., et al. (2013) | Australian Diabetes Foot Network: practical guideline on the provision of footwear for people with diabetes. J Foot Ankle Res 6(1): 6. | Guidelines. | Not specific ATSI |
| Browne, J., et al. (2014) | Feltman: evaluating the acceptability of a diabetes education tool for Aboriginal health workers. Aust J Prim Health 20(4): 319-322. | 'Feltman', a life-sized felt body showing the main organs involved in the digestion and metabolism of food, and the main parts of the body affected by diabetes. | No foot program |
| Burrow, S. and K. Ride (2016) | Review of diabetes among Aboriginal and Torres Strait Islander people. Australian Indigenous HealthInfoNet. | This review discusses the issues of prevention and management of diabetes,  and provides information on relevant programs, services, policies and strategies. | Review |
| Canuto, K., et al. (2017). | Aboriginal and Torres Strait Islander health promotion programs for the prevention and management of chronic diseases: a scoping review protocol. JBI Database of Systematic Reviews & Implementation Reports 15(1):10-14 | Identifies and describes the existing research on health promotion programs and activities focusing on modifying risk factors and/or improving the management of chronic diseases. | Review |
| Charles, J. (2015) | An evaluation and comprehensive guide to successful Aboriginal health promotion." Australian Indigenous Health Bulletin 16(1) | Examines and evaluates a report on an Aboriginal health promotion program on: its effectiveness in reducing injury, poor foot health, smoking, alcohol consumption and improved diet. | Not foot specific |
| Chung, F., et al. (2014) | Diabetes clinic attendance improves diabetes management in an urban Aboriginal and Torres Strait Islander population. Aust Fam Physician 43(11): 797-802. | Retrospective clinical audit of adult patients with type 2 diabetes who attended Winnunga Nimmityjah Aboriginal Health Service in Canberra including foot checks. | Not foot specific |
| Clement, Z. (2011). | Diabetic Foot Ulcer Management: Clinical and Cost Effectiveness of Vacuum Assisted Closure Therapy. Aboriginal and Islander Health Worker Journal 35(2): 5-8. | Systematic review. | Not ATSI |
| Connors, C. (2011). | Chronic disease in the Northern Territory (NT): Improving aboriginal health through a systems approach. Intern Med 41(s2). | Population systems approach to screening and management of chronic disease. | Review |
| Cooper, J., et al. (2007) | Partnership approach to Indigenous primary health care and diabetes: a case study from regional New South Wales. Aust J Rural Health 15(1): 67-70 | The Goorie Diabetes Complication and Assessment Clinic including foot checks. | Not foot specific |
| Coyle, M. E., et al. (2013) | Self- management activities in diabetes care: A systematic review. Australian Health Review 37(4): 513-522. | Self-management activities including adherence to medication, self-monitoring of blood glucose, dietary changes, physical activity and foot care. | Not ATSI |
| Cribbes, M. and K. Glaister (2007) | 'It's not easy' - caring for Aboriginal clients with diabetes in remote Australia. Contemporary Nurse: A Journal for the Australian Nursing Profession 25(1-2): 163-172 | Personal perspective of caring for clients with diabetes on a remote Aboriginal community in the Northern Territory. The purpose of the paper is to highlight the dilemmas faced by health care professionals as they endeavour to make a real change to the alarming health status of the Indigenous people of Australia. | Review |
| D'Abbs, P., et al. (2008) | Implementing a chronic disease strategy in two remote Indigenous Australian settings: A multi-method pilot evaluation. Australian Journal of Rural Health 16(2): 67-74 | Evaluation of framework for monitoring implementation of The North Queensland Indigenous Chronic Disease Strategy including foot checks. | Not foot specific |
| Davis, S., et al. (2015) | How good are routinely collected primary healthcare data for evaluating the effectiveness of health service provision in a remote Aboriginal community? Rural & Remote Health 15(4): 1-9. | Effectiveness of management system in the delivery of health services to the Fitzroy Valley in the Kimberley region of Western Australia. | No foot program |
| Eades, S., et al. (2014) | Baseline evidence practice gap for type 2 diabetes care among Aboriginal Australians in a cluster randomised controlled trial. BMC Health Serv Res 14(Suppl 2): P33-P33. | Examine the effectiveness of a tailored model in achieving adherence to best practice clinical guidelines for Type 2 diabetes in Aboriginal Community Controlled Health Organisations. | Not foot specific |
| Forbes, M. P., et al. (2013) | Impacts and outcomes of diabetes care in a high risk remote Indigenous community over time: implications for practice. Aust J Prim Health 19(2): 107-112. | Determines diabetes care processes and intermediate clinical outcomes in a remote  primary care service in 2009 compared with 2004 including foot checks. | Not foot specific |
| Gibson, O., et al. (2016) | The South Australian Aboriginal Diabetes Strategy 2017 – 2021. Adelaide, Wardliparingga Aboriginal Research Theme, South Australian Health and Medical Research Institute | State-wide response to diabetes and guide potential health care reforms for diabetes and related conditions including foot complications. | Review |
| Gibson, O. R. and L. Segal (2015) | Limited evidence to assess the impact of primary health care system or service level attributes on health outcomes of Indigenous people with type 2 diabetes: a systematic review. BMC Health Serv Res 15: 154 | Systematic review. | Review |
| Harch, S., et al. (2012) | Management of type 2 diabetes: A community partnership approach. Aust Fam Physician 41(1-2): 73-76. | Evaluation of a new model of partnership care using an audit cycle and including foot checks. | Not foot specific |
| Harris, C. and O. Curtis (2005) | Supporting Self- management of Diabetes in Aboriginal People Living with Diabetes through a 5 Day Residential Camp. Aboriginal and Islander Health Worker Journal 29(3): 4-11. | Developed, trialled and evaluated a culturally appropriate model of disease self-management in a group of Aboriginal people with established diabetes. | No foot program |
| Harvey, P. W., et al. (2013) | Chronic condition management and self-management in Aboriginal communities in South Australia: outcomes of a longitudinal study. Australian Health Review 37(2): 1-1. | Processes and outcomes of chronic condition management and self-management strategies implemented in three Aboriginal communities in South Australia. | No foot program |
| Heiss, E. (2000) | Aboriginal Diabetes Program. Aboriginal and Islander Health Worker Journal 24(5): 30-31. | Aboriginal Diabetes program with an aim to improve health care in Aboriginal and Torres Strait Islander people who have, or are at risk, of diabetes in the areas of access to medical care, control of diabetes  and education about healthy lifestyles. | Not foot specific |
| Hotu, C., et al. (2018) | Impact of an integrated diabetes service involving specialist outreach and primary health care on risk factors for micro- and macrovascular diabetes complications in remote Indigenous communities in Australia. Aust J Rural Health. | Integrated diabetes service involving specialist outreach and primary health care teams targeting risk factors for micro- and macrovascular diabetes complications in three remote Indigenous Australian communities. | No foot program |
| Hoy, W. E., et al. (2006) | Setting up chronic disease programs: Perspectives from Aboriginal Australia. Ethnicity and Disease 16(SUPPL. 2): S2-73 - S72-78. | Perspectives on setting up programs to improve management of hypertension, renal disease, and diabetes in high-risk populations in remote Australian Aboriginal settings. | No foot program |
| Jones, S., et al. (2011) | The foot book : a manual for Aboriginal health workers about common foot problems, how to recognise them and what to do about them (3^rd^ edition) | The foot book was originally designed for use as a reference book for Aboriginal Health Workers undertaking the foot care workshop designed by the Uni SA / Nunkuwarrin Yunti Foot Project Team. | Not foot specific |
| King, M., et al. (2013) | Issues that impact on Aboriginal health workers' and registered nurses' provision of diabetes health care in rural and remote health settings. Aust J Rural Health 21(6): 306-312. | Identify issues that compromise the clinical practice of rural and remote Aboriginal health workers and registered nurses who undertook an accredited Australian Diabetes Educators Association diabetes course. | No foot program |
| King, M. and K. Wilson (1998) | Aboriginal Projects Undertaken in South Australia. Aboriginal and Islander Health Worker Journal 22(6): 24-27 | Construction of a flip-chart entitled ‘Diabetes and Pregnancy’ and why Aboriginal health workers should consider undertaking an Australian Diabetes Educators course. | No foot program |
| Kit, J. A., et al. (2003) | Chronic disease self-management in Aboriginal communities: Towards a sustainable program of care in rural communities. Aust J Prim Health 9(2-3): 168-176 | Developed and trialled new program tools and processes for goal setting, behaviour change and self-management for Aboriginal people with diabetes. | No foot program |
| Kowanko, I., et al. (2012) | Chronic Condition Management Strategies in Aboriginal Communities: Final Report 2011. Adelaide, Flinders University and the Aboriginal Health Council of South Australia. | Final report of a project called ‘Chronic Condition Management Strategies in  Aboriginal Communities’ conducted during 2008-2011. | Not foot specific |
| Larkins, S., et al. (2015) | Responses of Aboriginal and Torres Strait Islander Primary Health-Care Services to Continuous Quality Improvement Initiatives. Front Public Health 3: 288 | Examined trends in quality of care for Indigenous primary health-care services participating in continuous quality  improvement cycles. | No foot program |
| Liaw, S. T., et al. (2011) | Successful chronic disease care for Aboriginal Australians requires cultural competence. Australian & New Zealand Journal of Public Health 35(3): 238-248 | Reviewed the literature to determine the attributes of culturally appropriate healthcare to inform the design of chronic disease management models for Aboriginal patients in urban general practice. | Review |
| Longstreet, D. A., et al. (2005) | Improving diabetes care in an urban Aboriginal medical centre. Aust J Prim Health 11(3): 25-31. | Improve the detection, monitoring, and medical care of Indigenous patients with diabetes in an urban Aboriginal medical centre, including foot checks. | Not foot specific |
| Mak, D. B., et al. (2004) | So far and yet so close: quality of management of diabetes in Australian and Canadian Indigenous communities." Aust J Rural Health 12(5): 206-209 | Compared quality of clinical management of Indigenous people with diabetes in remote areas of Australia and Canada. | Review |
| Maple-Brown, L. J., et al. (2004) | Diabetes care and complications in a remote primary health care setting. Diabetes Res Clin Pract 64(2): 77-83 | A population survey of risk factors for diabetes and cardiovascular disease and diabetes prevalence. | Not foot specific |
| Marley, J. V., et al. (2012) | Quality indicators of diabetes care: an example of remote-area Aboriginal primary health care over 10 years. Med J Aust 197(7): 404-408 | Described service characteristics of Derby Aboriginal Health Service and documented diabetes management activities and intermediate clinical outcomes for Aboriginal patients with type 2 diabetes. | No foot program |
| Matthews, V., et al. (2014) | Duration of participation in continuous quality improvement: a key factor explaining improved delivery of Type 2 diabetes services. BMC Health Serv Res 14: 578 | Examined variation in quality of Type 2 diabetes service delivery in over 100 Aboriginal and Torres Strait Islander primary health care centres participating in a wide-scale CQI project over a decade. | Not foot specific |
| McCalman, J., et al. (2016) | The effectiveness of implementation in Indigenous Australian healthcare: an overview of literature reviews. Int J Equity Health 15(47). | Analyses Aboriginal and Torres Strait Islander Australian health implementation reviews to examine the research question: What is the effectiveness of implementation, as reported in the Indigenous Australian health implementation literature? | Review |
| McDermott, R. A., et al. (2001) | Improving diabetes care in the primary healthcare setting: a randomised cluster trial in remote Indigenous communities. Med J Aust 174(10): 497-502. | Evaluates a system for improving diabetes care in remote indigenous communities. | Not foot specific |
| McDermott, R., et al. (2003) | Sustaining better diabetes care in remote indigenous Australian communities. BMJ 327(7412): 428-430. | Three year follow up clinical audit of 21  primary healthcare centres where local indigenous health workers used registers,  recall and reminder systems, and basic diabetes care plans, supported by a specialist outreach service. | Not foot specific |
| McDermott, R. A., et al. (2004) | Diabetes care in remote northern Australian Indigenous communities. Med J Aust 180(10): 512-516 | Clinical audit from diabetes registers in 21 remote primary healthcare centres. | Not foot specific |
| McDermott, R. and L. Segal (2006) | Cost impact of improved primary level diabetes care in remote Australian indigenous communities. Aust J Prim Health 12(2): 124-130 | Reports direct costs and downstream savings of improved quality of diabetes services, compared to usual care, in the primary care setting in a high risk remote Indigenous Islander population. | Not foot specific |
| McDermott, R. A., et al. (2007) | Diabetes in the Torres Strait Islands of Australia: better clinical systems but significant increase in weight and other risk conditions among adults, 1999-2005. Med J Aust 186(10): 505-508 | Assessed changes in clinical indicators of adults diagnosed with diabetes. | Not foot specific |
| McDermott, R. A., et al. (2015) | Community health workers improve diabetes care in remote Australian Indigenous communities: results of a pragmatic cluster randomized controlled trial. BMC Health Serv Res 15(1): 1-8. | Evaluated the effectiveness of a community-based health-worker led case management approach to the care of Indigenous adults with poorly controlled type 2 diabetes in primary care services in remote northern Australia. | Not foot specific |
| McNamara, B. J., et al. (2011) | Type 2 diabetes in Indigenous populations: quality of intervention research over 20 years. Prev Med 52(1): 3-9. | Evaluates the quantity and methodological quality of published intervention research on Type 2 and gestational diabetes in the Indigenous populations of Australia, Canada, New Zealand, and the United States from 1989 to 2008. | Review |
| Mobbs, R., et al. (2003) | The chronic disease self-management project at Katherine West Health Board Aboriginal Corporation in the Northern Territory: A report of the first year. Aust J Prim Health 9(2-3): 160-167 | One year review of the Chronic Disease Self-Management Demonstration Project (CDSM) which commenced in April, 2002. | No foot program |
| Nguyen, H. D., et al. (2016) | Management of diabetes in Indigenous communities: lessons from the Australian Aboriginal population. Intern Med J 46(11): 1252-1259. | Discusses strategies to address the large disparities in life expectancy between Indigenous and non-Indigenous Australians | Review |
| Panaretto, K. S., et al. (2013) | Prevention and management of chronic disease in Aboriginal and Islander Community Controlled Health Services in Queensland: A quality improvement study assessing change in selected clinical performance indicators over time in a cohort of services. BMJ Open 3(4). | Evaluated clinical healthcare performance  in Aboriginal Medical Services in Queensland. | No foot program |
| Pearce, S., et al. (2005) | The Better Living Diabetes Project. Aboriginal and Islander Health Worker Journal 29(1): 4-6. | Aim: to reduce the health impact of  diabetes by educating sufferers and their families and facilitating change in relation to diabetes and lifestyle. Has a focus on education (of project participants and health workers) and delivery of clinical support. | Not foot specific |
| Regan, T., et al. (2017) | Comparison of Two Sources of Clinical Audit Data to Assess the Delivery of Diabetes Care in Aboriginal Communities. Int J Environ Res Public Health 14(10) | Determined the concordance between data extracted from two Clinical Decision Support Systems regarding diabetes testing and monitoring at Aboriginal Community Controlled Health Services in Australia. | No foot program |
| Schierhout, G., et al. (2016) | Improvement in delivery of type 2 diabetes services differs by mode of care: a retrospective longitudinal analysis in the Aboriginal and Torres Strait Islander Primary Health Care setting. BMC Health Serv Res 16(1): 560 | Clinical audit of clients with Type 2 diabetes collected as part of a wide-scale CQI program implemented between 2005 and 2014 in 162 Aboriginal and Torres Strait Islander health centres. | Not foot specific |
| Schoen, D. E., et al. (2016) | Improving rural and remote practitioners' knowledge of the diabetic foot: findings from an educational intervention. J Foot Ankle Res 9: 26 | High Risk Foot intervention including an electronic risk tool with clinical decision  Support, Multidisciplinary Foot Ulcer Telehealth Clinic, Aboriginal diabetes foot care education brochures and movies. | Not just ATSI |
| Si, D., et al. (2005) | Assessing health centre systems for guiding improvement in diabetes care. BMC Health Serv Res 5: 56. | Assesses the status of systems for chronic illness care in Aboriginal community health centres. | Combined |
| Si, D., et al. (2006) | Aboriginal health workers and diabetes care in remote community health centres: a mixed method analysis. Med J Aust 185(1): 40-45 | Three year follow up to assess the effect of employing Aboriginal health workers (AHWs) on delivery of diabetes care in remote community health centres. | Combined |
| Si, D., et al. (2008) | Describing and analysing primary health care system support for chronic illness care in Indigenous communities in Australia's Northern Territory - use of the Chronic Care Model. BMC Health Serv Res 8: 112-112 | A mail-out survey to collect information on material, financial and human resources relating to chronic illness care in 12 Indigenous communities in Australia's  Northern Territory. | Review |
| Si, D., et al. (2010) | Assessing quality of diabetes care and its variation in Aboriginal community health centres in Australia. Diabetes Metab Res Rev 26(6): 464-473 | Clinical audits during 2005 -2009 to assess quality of diabetes care and its variation among Aboriginal community health centres in Australia. | Not foot specific |
| Simmons, D. (2003) | Impact of an integrated approach to diabetes care at the Rumbalara Aboriginal Health Service. Intern Med J 33(12): 581-585 | Describes the effectiveness of an integrated primary–secondary care diabetes clinic on metabolic control among Indigenous patients in a rural community. | No foot program |
| Spurling, G. K., et al. (2013) | Implementing computerised Aboriginal and Torres Strait Islander health checks in primary care for clinical care and research: A process evaluation. BMC Medical Informatics and Decision Making 13(1) | Describes the rationale, implementation and anticipated benefits of computerised Aboriginal and Torres Strait Islander health  checks in one primary health care setting. | Not foot specific |
| Stoneman, A., et al. (2014) | Quality improvement in practice: improving diabetes care and patient outcomes in Aboriginal Community Controlled Health Services. BMC Health Serv Res 14: 481. | Describes the evaluation of care of patients with type 2 diabetes mellitus and features of effective continuous quality improvement in ACCHSs in the remote Kimberley region of north Western Australia. | Not foot specific |
| Wong, M., et al. (2005) | Perspectives on clinic attendance, medication and foot-care among people with diabetes in the Torres Strait Islands and Northern Peninsula Area. Aust J Rural Health 13(3): 172-177. | Descriptive study collecting qualitative data  in three key areas of diabetes self-care, namely attending appointments at the clinic, monitoring blood glucose levels and taking medication and foot-care. | Review |
| Wardliparingga Aboriginal Research Unit at SAHMRI (South Australian Health and Medical Research Institute) | The Aboriginal Diabetes Study. <https://aboriginaldiabetes.com/> | Aims to improve the delivery of diabetes care to Aboriginal people and find solutions to better treat Type 2 Diabetes and prevent complications. Commenced in June 2015. Due for completion in December 2019. | Not foot specific |
